# Hyperuricemia in Patients With Coronary Artery Disease and Its Association With Disease Severity

**DOI:** 10.7759/cureus.17161

**Published:** 2021-08-13

**Authors:** Jaskamal Padda, Khizer Khalid, Abdulelah H Almanie, Hussam Al Hennawi, Krutagni Adwait Mehta, Ransirini Wijeratne Fernando, Sandeep Padda, Ayden Charlene Cooper, Gutteridge Jean-Charles

**Affiliations:** 1 Internal Medicine, JC Medical Center, Orlando, USA; 2 Internal Medicine, AdventHealth & Orlando Health Hospital, Orlando, USA

**Keywords:** cad: coronary artery disease, uric acid, hyperuricemia, cardiovascular disease, colchicine

## Abstract

The biochemical background of coronary artery disease (CAD) has been intensively explored in the past several decades. Previous clinical investigations have demonstrated the association of non-traditional risk factors, such as hyperuricemia, with CAD. Studies have shown that increased serum uric acid (SUA) was associated with an increased risk of adverse cardiovascular (CV) outcomes in patients with CAD. While the exact pathophysiological mechanisms leading to increased risk are still unknown, it has been postulated that hyperuricemia leads to endothelial dysfunction, oxidative metabolism, and platelet adhesiveness and aggregation, leading to CAD. Moreover, previous studies have shown that hyperuricemia is an independent risk factor for CAD. However, the correlation between high SUA levels and the severity of CAD remains unclear. The purpose of this review was to elucidate the association of hyperuricemia to CAD severity and to determine the effect of urate-lowering therapy (ULT) on CAD. A search of PubMed up to June 24, 2021, was carried out by the reviewers. From the findings, hyperuricemia stands as an independent risk factor for CAD, and CAD patients treated with ULT had improved CV outcomes and reduced mortality. Therefore, while SUA level is valuable in predicting an augmented risk of CAD and anticipating worse outcomes, ULT has promising cardioprotective effects.

## Introduction and background

Hyperuricemia has been described to be associated with cardiovascular (CV) diseases, including hypertension, stroke, and coronary artery disease (CAD). However, the mechanism of how serum uric acid (SUA) is associated with CAD has not been elucidated [1]. The exact role of SUA as an independently standing coronary heart disease risk factor is still controversial [2].

Moreover, since other comorbidities frequently exist with CAD, it is considered complex to differentiate the SUA function in this instance. CAD is considered to be the leading cause of death in the elderly population, with an incidence rate greater than 80% occurring in patients older than 65 years [1]. Pathophysiological mechanisms associating hyperuricemia with CAD have been established, with SUA being a stimulant to oxidative stress, inducing the production of oxygen free radicals and adhesion of platelets. These processes result in inflammatory reactions and dysfunction of the endothelium, which may explain the correlation between hyperuricemia and CAD [3].

Zuo et al. have concluded that hyperuricemia creates a potential risk for CAD and its associated mortality [3]. Although it has been stated that there are both safe and effective measures that can be used to lower SUA levels and therefore reduce cardiac disease, these still have to be explored further. In highlighting the cruciality of the SUA levels in association to CAD, Kim, et al. have noticed that the risk of death due to CAD is elevated by 12% for each 1 mg/dl increase of SUA [2].

## Review

Coronary artery disease

CAD is a pathologic condition that occurs when there is inadequacy in both blood supply and oxygen to the myocardial tissue. This condition results from luminal plaque formation in the coronary arteries, leading to blood flow hindrance, further leading to arterial occlusion. CAD has been the leading cause of mortality, both worldwide and in the United States. Moreover, it reached its peak of incidence in the 1960s and remained to be the ultimate leading cause of mortality [4]. In the United States, 16.8 million are affected by CAD [5]. Furthermore, it accounts for almost 610,000 deaths each year in the United States [6].

The first manifestation of CAD is chronic stable angina, which occurs in 50% of the patients. This phenomenon is a manifestation of an arterial obstruction of at least one large epicardial artery. Reduction of the associated symptoms and ischemia in order to avoid myocardial infarction (MI) and consequent death is the highlight of CAD management [5].

Possible risk factors for CAD are further classified into modifiable and non-modifiable risk factors. The modifiable factors include hypertension, hyperlipidemia, obesity, diabetes mellitus, and smoking. On the other hand, non-modifiable risk factors include gender, ethnicity, family history, and age of the patient with increased prevalence in patients greater than 35 years old in all genders [6].

Hyperuricemia

Hyperuricemia is defined as extracellular fluid supersaturation with abnormally elevated levels of uric acid, which can be caused by different pathways such as genetic disorders, overproduction of uric acid, and decreased renal excretion [7]. The most common cause of hyperuricemia is renal underexcretion and this type of hyperuricemia is considered primary or idiopathic [7]. The cut-off serum level of hyperuricemia is > 7mg/ dL, which can be precipitated by a diversity of risk factors, for instance, excessive consumption of a purine-rich diet, chemotherapy, and genetic disorders [8]. In humans, purine metabolism through the process of oxidation will yield in the production of uric acid [9]. Xanthine oxidoreductase is considered the catalyst enzyme participating in the process of converting hypoxanthine to xanthine and then further to uric acid [8]. Regardless of the evidence-based correlation between hyperuricemia and various types of disorders, for example, CAD, heart failure, and chronic kidney disease, there are no obvious guidelines regarding the management of asymptomatic hyperuricemia [10]. Gout is a metabolic disorder that is characterized by the deposition of monosodium urate crystals in joint spaces, resulting in acute or recurrent inflammatory arthritis [11]. Since the correlation between elevated SUA levels and gout has been established, the symptomatology of hyperuricemia as an abnormal finding can range from asymptomatic to intense arthritis [11]. In cases where gout is left untreated, it can result in a chronic course of increased flare-ups, development of anatomical joint damage, and tophi [12]. The treatment of gout aims at reducing the levels of SUA. Elevated SUA can be caused and influenced by various factors like aging, dietary factors, and lifestyle [12]. The saturation point of the SUA level can be attributed as 6.8 mg/dL [12]. As a means of therapeutic approach, the treatment and prevention of gouty arthritis is implemented via pharmacologic intervention. Patients should receive urate-lowering therapy (ULT), with the goal of SUA level <6.0 mg/dL [12].

Association between hyperuricemia and CAD

Hyperuricemia has been found in CAD, which is confirmed by angiography in the majority of population data, which is independent of other CV risk factors that are considered confounding. Hyperuricemia showed an independent trend in the severity of CAD. Gender variation in levels of uricemia was not found [13].

Some studies, including the Framingham Heart Study and the Atherosclerosis Risk in Communities (ARIC) study, have shown no association between uric acid and CAD [14-15]. However, numerous recent papers have suggested some association. Hiyamuta et al. did a case-control study on 1029 patients (87% male) who were undergoing coronary angiography. They divided them into four groups according to arteriographic findings [16]. Uric acid was not found to have an independent association with CAD or its severity. A few other studies like Kotake and colleagues, Tuttle and colleagues, etc. also found a linear relationship of SUA levels with CAD [17-18].

Fang and colleagues conducted a cross-sectional follow-up study, which has a representative sample of the United States adult population and has shown an increased level of association of increasing uric acid levels with CAD. Hazard ratios for men and women were determined after the adjustment of risk factors such as age, body mass index, cholesterol levels, smoking status, etc. [19-20]. High levels of SUA have been found as a strong risk factor for adverse outcomes and mortality in severe CAD (with >70% stenosis in coronary angiography) [21].

The study was done in Framingham, Massachusetts to examine uric acid serum levels in relation to death from all-cause, CAD, and incidence of CAD. A total of 117,376 person-years were examined to note 617 attributed to CAD, 1460 deaths from all causes, and 429 CV deaths. The study did not find a causal role of SUA in CAD development, death from all causes, or death from cardiovascular diseases [15].

Hyperuricemia has not only been found to have an independent association with the development of CAD, but there is also complex interrelations with many other independent CV risk factors such as diabetes, obesity, metabolic syndrome, and chronic kidney disease [13]. This makes it hard to confirm independent studies to investigate a firm, independent association of uric acid levels with CAD. Although the association of hyperuricemia with CAD has been found in many studies, some studies have also shown severity association with CAD [13].

A multivariate study on 771 participants with 37% participants having high uric acid levels (defined as >6 mg/dl in females and >7 in males) showed high uric acid levels as an independent risk factor in CAD severity [12]. Incidence of CAD is rare but rising in the age group less than 35 years. This study has shown hyperuricemia as an independent risk factor for acute coronary syndrome in the 18-35 age group [22].

The prognostic value stays unclear with CAD with heavy atherosclerotic burden in all three vessels. A cohort study of 8529 patients was conducted to determine the all-cause death as an endpoint, propensity score matching was done to compare two cohort groups. Hyperuricemia was present in 14.2% of total patients. At baseline hyperuricemia, patients had more comorbidities. During 7.5 years of median follow-up, hyperuricemia patients had been associated with significantly more deaths (39.11% more death, p<0.001) compared with normouricemic patients [23]. Hyperuricemia was associated with a higher risk of mortality in a multivariate analysis (1.3 hazard ratio [HR] with 1.15 to 1.53 95% confidence interval [CI], p<0.001). Also, similar results were found in propensity score matching (1.3 HR, 1.11 to 1.61 95% CI, p<0.003). This association was found to be consistent in different subgroups except between age subgroups. This study also found a statistically significant association of hyperuricemia with CAD mortality [23]. Such disparity in study results may be due to the differences between genetic background and lifestyle, which affects uric acid levels. Uric acid level variability can also be due to various study populations and differences in sample size [24-25]. Table 1 discusses the association between hyperuricemia and CAD [15-18,22-23].

**Table 1:** Association between hyperuricemia and CAD N/A - Not Available, CV - Cardiovascular, SUA - Serum Uric Acid, CAD - Coronary Artery Disease, ACS - Acute Coronary Syndrome

Referenced Trial	Sample Size	Study Participants	Mean Uric Acid Levels	Primary Outcomes	Objective	Conclusion
The Framingham Heart Study (1999) Massachusetts [15]	6763	Mean age of 47 years old; 3075 men, 3688 women	Men 379 umol/L ± 76; women 285 umol/L ± 69	617 coronary heart disease events; 429 CV disease deaths; 1460 deaths from all causes	To examine the relation of SUA level to incident coronary heart disease, death from CV disease, and death from all causes	Uric acid does not have a causal role in the development of coronary heart disease, death from CV disease, or death from all causes
Hiyamuta K, et al. (1990) Japan [16]	1029; 644 patients with previous MI; 385 patients with angina pectoris	892 men and 137 women	5.91 mg/dl ±1.54	SUA and smoking are considered to be risk factors for patients	To analyze the relationship between coronary risk factors and arteriographic features of coronary atherosclerosis	Smoking and hyperuricemia are strongly correlated with all types of coronary atherosclerosis
Kotake H, et al. (1992) [17]	97	40 postmenopausal women, 57 men	N/A	The mean value of SUA increased as the number of stenosed coronary arteries increased (greater or equal to 50% stenosis)	Relation between SUA and angiographically defined CAD in postmenopausal women	Elevated SUA levels suggest the prevalence of severe coronary artery stenosis in postmenopausal women
Tuttle KR, et al. (2001) [18]	277	82 women, 195 men	5.4 mg/dL for women; 6.3 mg/dL for men	Linear elevation of SUA in women; no change in men	To determine gender differences in the risk factors for CAD	SUA is linearly related to CAD severity in women, not in men
Lv S, et al (2019) [22]	771	Mean age of 31.6	7.0 mg/dL in men; 6.0 mg/dL in women	Gensini score identified hyperuricemia to be an independent risk factor for CAD severity	Assess the relationship between hyperuricemia and CAD severity	Hyperuricemia was shown to be an independent risk factor for CAD severity in young adults with ACS
Zhang C, et al. (2018) China [23]	8529	1749 female, 6780 males	322 umol/L in men; 281.1 umol/L in women	Hyperuricemia is associated with an increased risk of mortality	To evaluate the prognostic value of hyperuricemia in patients with severe CAD	Individual increments in SUA have been shown to increase mortality risk in patients with CAD

Effect of anti-gout medications on CAD

Current evidence illustrates the negative association of hyperuricemia and gout, which were shown to be independently associated with an elevated risk of heart diseases, including CAD, congestive heart failure, and fatal CV events [26]. In fact, known active gout patients who present with acute MI were shown to have worse survival outcomes in comparison to patients on gout treatment [27]. Moreover, the URic acid Right for heArt Health Study Group (URRAH) proved the independent link between SUA and severe cases of MI [28]. Of note, not all ULTs have been shown to improve CV outcomes, however, colchicine, a well-known gout medication, has shown a long-term reduction in fatal CV events in patients following recent MI and known stable ischemic heart disease patients.

The Colchicine Cardiovascular Outcomes Trial (COLCOT) demonstrated a 1.6% absolute mortality reduction from CV causes, resuscitated cardiac arrest, recurrent MI in patients when treated with low-dose colchicine (0.5 mg once daily) within 30 days of MI (Mean of 13.5 days after MI) compared with the placebo group (HR 0.77, 95% CI 0.61-0.96; p = 0.02) [29]. At a cellular level, in vitro application of colchicine was shown to inhibit platelet activity; reflecting decreased rates of in-stent stenosis and stabilization of coronary plaques following acute coronary syndrome in vivo [30]. Notably, a smaller infarct size (18.3 vs. 23.2 mL/1.73 m2 in placebo; p = 0.019) was demonstrated following the use of colchicine in ST-elevation MI within 12 h of the presentation along with standard percutaneous coronary intervention [31]. Additionally, two retrospective studies demonstrated a significant reduction of CV events in patients with gout managed with colchicine compared to people who were not on treatment [32-33]. Emerging evidence is advocating the cost-effectiveness and safety profile of colchicine use among coronary vascular disease patients and larger trials with longer follow-up intervals are warranted in certain patient subgroups to emphasize associated benefits of colchicine therapy as add-on therapy or for secondary prevention of CV events.

Similar to colchicine, the ULT allopurinol has varying CV effects. According to a randomized control trial, allopurinol demonstrated increased exercise capacity, time to ST depression, and time to symptom onset (chest pain) in 65 patients with CAD [34]. Cardioprotective effects of allopurinol were also postulated in larger population-based and different case-control studies. In particular, de Abajo et al. brought to light possible protective effects of allopurinol following longer duration and higher dosage of treatment (> 300 mg compared with < 300 mg) [35]. Similarly, an observational study suggested protective effects of allopurinol for MI in elderly people supporting earlier Medicare claims [36]. Allopurinol benefits are not only limited to CAD; the possible blood-pressure reduction was also evident. Compared to placebo/no treatment, allopurinol ≤ 300 mg daily was associated with insignificant reduction of major adverse cardiovascular events (MACE) and mortality, however, reduced risks of hypertension (Odds Ratio (OR) 0.54, 95% CI 0.37-0.80) and total events (OR 0.60, 95% CI 0.44-0.82) were observed [37]. Xanthine oxidase (XO) inhibition was also shown to enhance endothelial function and subsequent vasodilation and larger blood flow in patients with HF achieved by allopurinol in normouricemia and hyperuricemia patients suggesting a crucial effect of oxidative stress inhibition in such cohort [38].

Febuxostat, another XO inhibitor, demonstrated questionable cardioprotective effects. In fact, patients treated with febuxostat for gout reported a greater incidence of CV events compared to allopurinol treatment, although results were not statistically significant according to early trials [39-42]. The CARES trial, which was mandated by the United States Food and Drug Administration (USFDA), compared gout treatment with febuxostat or allopurinol for a median of 32 months. Although results have shown no significant difference in terms of the primary endpoint of composites, including CV death, non-fatal MI, non-fatal stroke, and unstable angina requiring revascularization. Nevertheless, the febuxostat group demonstrated significant CV mortality compared to allopurinol (HR 1.34, CI 95% 1.03-1.73) [43]. For this reason, the USFDA announced a black box warning for the use of febuxostat in patients with gout who showed no response to maximum allopurinol therapy [44].

## Conclusions

As the worldwide clinical and financial burden of CAD is rapidly rising, we have attempted to analyze the evidence suggesting a correlation between hyperuricemia and CAD. Because of the effects of SUA on atherogenesis, we hypothesized high levels of SUA were associated with the severity of CAD. Our findings suggested that high levels of SUA were significantly associated with the severity of CAD, apart from two studies that did not show a correlation. Whether the lack of correlation in those studies was due to confounding was unclear. Our findings also suggested that ULT (colchicine and allopurinol) was associated with cardioprotective effects and improved CV outcomes.

In addition to the evaluation of conventional risk factors in daily clinical practice, measurement of the SUA level might be an important, simple, and cost-effective vascular risk marker, which is routinely measured in clinical practice that can provide significant prognostic benefits in terms of global CV risk and management of patients; further, high levels of SUA levels may become surrogate markers of CAD severity. Moreover, ULT can gain recognition in the management of CAD in the future.
